# Does robotic arm-assisted total knee arthroplasty have a role to play in large deformities?

**DOI:** 10.1051/sicotj/2024046

**Published:** 2024-11-21

**Authors:** Pierre-Henri Vermorel, Carlo Ciccullo, Luca De Berardinis, Antonio Pompilo Gigante, Thomas Neri, Rémi Philippot

**Affiliations:** 1 Department of Orthopaedic Surgery, University Hospital Centre (Saint Etienne) Avenue Albert Raimond 42270 Saint-Priest-en-Jarez France; 2 Inter-University Laboratory of Human Movement Science, University Lyon – University Jean-Monnet Saint-Étienne Saint-Étienne France; 3 Clinical Orthopaedics, Department of Clinical and Molecular Sciences, Università Politecnica delle Marche 60121 Ancona Italy

**Keywords:** Knee, Total knee arthroplasty, Robotic-assisted total knee arthroplasty, Knee large deformity

## Abstract

*Background*: Total knee arthroplasty (TKA) for patients with a large preoperative deformity (more than 10° varus or valgus) remains a challenge leading to a high rate of outliers, unsatisfactory functional results, or early prosthetic loosening. Robotic arm-assisted TKA (RATKA) has shown improvements in implant positioning accuracy. This study aimed to assess RATKA implant positioning accuracy and functional results at one year postoperative for patients with a large preoperative deformity. *Methods*: From November 2019 to July 2022, 500 RATKA were performed. About 74 patients with more than 10° of varus or valgus global deformity were included. Each patient received a semi-constrained implant. The difference between the valgus or varus value planned intra-operatively and the varus or valgus measured on one-year postoperative X-rays has been assessed. Functional outcomes (VAS, range of motion, KOOS) have also been evaluated. *Results*: For varus, the mean difference was 0.54 ± 1.21°, all patients (100%) had a difference of less than 3° at one-year post-operative. For valgus, the mean difference was 0.63 ± 1.29°, most patients (92%) had a difference of less than 3° at one year postoperative. Overall, 98.6% (*n* = 73) of cases had a difference of less than 3° at one-year postoperative. The mean VAS was 1.6 ± 1.4 [1;4]. Mean flexion was 132 ± 7.6° [100;145]. A total of 69 patients (93%) had a good or excellent KOOS score (KOOS total > 70) at one year post-operative. *Conclusion*: For large preoperative deformities, RATKA provides a high degree of accuracy in implant positioning, permitting it to fit the desired alignment without compromising knee stability, and giving the possibility of using semi-constrained implants. At one year postoperative, functional results are encouraging and most patients have recovered an optimal range of motions.

## Introduction

Total knee arthroplasty (TKA) is one of the most frequent procedures performed in orthopaedic surgery (110 thousand procedures in France in 2018). The number of cases is expected to increase by 30.8% to 152.8% by 2050 [1].

Dissatisfaction rate after TKA is between 20% and 30% [2, 3]. Several reasons contribute to these results. Large postoperative residual deformity or mismatch between planned and postoperative implant positioning leads to unsatisfactory results. The presence of a large pre-operative deformity (>10° varus or valgus) may complexify the surgical act (bone and soft tissue procedures) and have a negative impact on the result [4]. Constraints implants can also be used for compensating a ligament balancing failure. Still, they present a higher risk of poor functional results, bone loosening and a poorer long-term survival rate.

Despite technical progress, a difference of more than 3° between the planned frontal alignment and the postoperative alignment still occurs in 32% of cases with conventional instrumentation. This rate is higher for patients presenting pre-operative large deformity (>10°) [5, 6].

To increase the safety of implant positioning, successive studies have been carried out on computer-assisted navigation and robotic surgery. In the 1990s, under the impetus of the Alpes-Grenoble University teams, navigated and image-guided surgery was developed [7]. Studies have demonstrated its effectiveness with a reduction of post-operative malalignment from 32% to 9% [8, 9].

More recently, robotic arm-assisted total knee arthroplasty (RATKA) has been developed to improve TKA procedures. Some cadaveric studies have already shown an improvement in bone cut accuracy with RATKA in comparison with conventional instrumentation [10]. Marchand et al. showed that 100% of patients undergoing RATKA for an initial deformity <7° had a final alignment in the frontal plane <3°, compared with 91% after navigation and 68% when a conventional instrumentation was used [11]. A meta-analysis by Lei et al. also shows RATKA superiority in comparison with navigation and patient-specific instrumentation (PSI) in terms of bone cut accuracy and implant positioning [12]. To our knowledge, no study has assessed RATKA outcomes for patients with a preoperative frontal large deformity defined by a varus or valgus value of more than 10°.

This study aimed to assess RATKA implant positioning accuracy for patients presenting a preoperative frontal large deformity. The secondary outcome is to evaluate functional and clinical outcomes for this population at one year postoperative.

We hypothesize that RATKA improves implant positioning accuracy for patients with a large preoperative deformity (varus or valgus value >10°) in comparison to conventional instrumentations.

## Materials and methods

### Inclusion

Patients were included from November 2019 to July 2022. RATKAs were performed by four senior surgeons.

Inclusion criteria were: (1) Patients with a varus or valgus value of more than 10° on the pre-operative long leg standing radiograph; (2) TKA performed with CT-based Mako Stryker^®^ Robotic assistance; (3) Skeletal maturity.

We have excluded patients in case of prosthetic revision surgery. The study did not require ethical approval. All patients provided their informed consent to the use of medical records and personal data at the admission.

### Study design

All patients underwent a pre-operative clinical examination to assess range of motion (ROM), morphotype, location of pain and ligament laxity in flexion and extension. A pre-operative knee X-ray assessment (long-leg standing radiographs of the lower limbs, bilateral AP, lateral, schuss, valgus and varus stress, and patella-femoral X-rays) has been done for each patient. A pre-operative CT scan was also performed to generate a three-dimensional model of the knee required for the Mako^®^ procedure.

TKA was performed with a medial parapatellar trans-tendinous approach. No bone procedures were performed to ensure ligament balance. Ligament releases were performed when ligament balance could not be achieved while preserving a planned alignment ranging between 3° valgus and 6° varus. All patients received a posteriorly stabilized STRYKER Triathlon^®^ prosthesis (implant with the lowest degree of constraint available in our centre at the time). Cementation of the implants was carried out only in cases of low bone density which could have compromised primary stability. Patellar arthroplasty was indicated in cases of pre-operative patellar pain, total absence of cartilage on the patellar articular surface, or patellar maltracking after positioning of the tibial and femoral components. Insufficient patellar thickness (predicted thickness <13 mm after resurfacing) contraindicated patellar prosthesis. The tourniquet was inflated only for the cementing phase if performed. A drainage system was used for all patients and removed the day after surgery.

**Figure 1:**
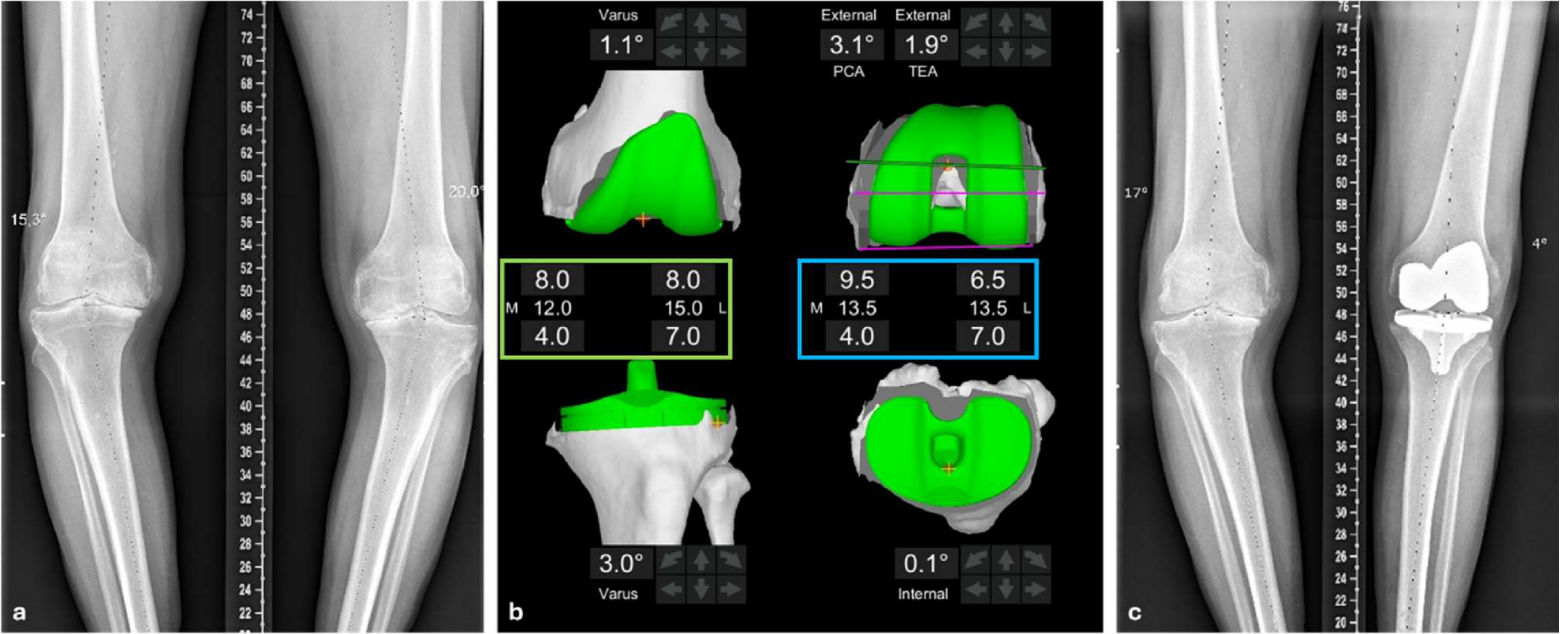
Management of a patient with a bilateral knee osteoarthritis with large deformity. a: preoperative long leg standing X-rays showing a varus deformation of 20°. (b) Intra-operative Mako settings. Coronal femoral component positioning is set to restore the native joint surface. Coronal tibial component positioning is set to obtain an equal medial and lateral extension gap. The green box represents the value of the distal femoral cuts (values at the top of the box), and the proximal tibia cuts (values at the bottom of the box). The middle values represent the total cut thickness. For this case, femoral varus is set at 1.1° and tibial varus is set at 3° to achieve this goal. In the axial plane, femoral component is positioned to align with the trochlear groove while having an equal medial and lateral flexion gap. The blue box represents the thickness of the posterior femoral cuts (values at the top of the box) and the proximal tibial cuts (values at the bottom of the box). The middle values represent the total cut thickness. For this case, femoral component rotation is set at 3.1° relative to the PCA (Posterior Condyle Axis) or 1.9° relative to the sTEA (surgical Trans-Epicondylar Axis). (c) One-year postoperative long-leg standing X-rays showing a global varus value of 4°.

Implants were positioned according to a patient-specific alignment close to the “restricted kinematic alignment” philosophy, limiting residual deformity to 6° varus and 3° valgus [13]. Ligament balance had to be equivalent in the medial and lateral knee compartments, in extension and at 90° flexion (Figure 1).

Follow-up was performed at 45 days to ensure good scar healing, absence of knee extension deficit, and knee flexion greater than 90°. A clinical check-up was then carried out at 3 months, 6 months, one year and two years post-surgery. A complete radiological check-up was performed one-year post-op. Long-leg standing X-rays were used to assess varus/valgus value at one year [14, 15].

KOOS functional score and ROM were recorded at 6-month, 1-year and 2-year postoperative.

### Outcomes assessment

We assessed the difference between the global varus or valgus value planned intra-operatively using the Mako^®^ system and the global varus or valgus value measured on the long leg standing X-rays at one year post-operative. Patients were considered as outliers if the difference was >3°.

Assessment of ROM, VAS, and KOOS was carried out at one year postoperative.

### Statistics

All statistical analyses were performed with SPSS Statistic^©^ software (SPSS Inc, IBM^©^, Armonk 151 NY, USA) version 28. The independent *t*-test was used to compare quantitative variables according to the normal distribution. A value of *p* < 0.05 was used to consider a difference as statistically significant.

## Results

### Population

A total of 500 patients underwent a CT-based RATKA, 426 patients were excluded because of a varus or valgus value of less than 10°. A total of 74 patients were analysed. The mean follow-up was 20 months ±4 [12;31] (Table 1).

**Table 1:** Patient characteristics.

Characteristics	
Age, y, mean, ± SD [range]	70 ± 10 [45;84]
BMI, mean, ± SD [range]	27.62 ± 4.9 [24;35]
Sex	
Female *n* (%)	31 (42%)
Male *n* (%)	43 (58%)
Morphotype	
Varus *n* (%)	62 (84%)
Valgus *n* (%)	12 (12%)
Side	
Right *n* (%)	34 (46%)
Left *n* (%)	40 (54%)
Implant fixation *n* (%)	
Femur cementless	57 (77%)
Femur cemented	17 (23%)
Tibia cementless	57 (77%)
Tibia cemented	17 (23%)
Polyethylene thickness *n* (%)	
9 mm	52 (70%)
11 mm	12 (30%)
Patella arthroplasty *n* (%)	
Yes	17 (23%)
No	57 (77%)
Pre-op flexion (degree), mean, ±SD, [range]	124 ± 15 [94;140]
Pre-op extension (degree), mean, ±SD, [range]	3.85 ± 6.95 [−12;19]

BMI: Body Mass Index (kg/m²), SD: Standard Deviation.

### Implant positioning accuracy

#### Varus

The mean varus was 12.7 ± 3.3° on preoperative radiographic measurement. The mean intra-operative planned varus was 3.9 ± 1.8° and 1-year postoperative mean varus was 3.3 ± 1.9°. The mean difference between planned varus and 1-year post-operative varus was 0.6 ± 1.1°. All patients (100%) had a difference between planned varus and 1-year post-operative varus of less than 3°.

#### Valgus

The mean valgus was 12.6 ± 2.5° on preoperative radiographic measurement. The mean intra-operative planned valgus was 0.6 ± 1.7° and the 1-year postoperative mean varus was 1.1 ± 2°. The mean difference between planned valgus and 1-year post-operative values was 0.5 ± 1.6°. 92% (*n* = 11) of cases had a difference between planned valgus and 1-year post-operative valgus of less than 3°.

Overall, 98.6% (*n* = 73) of cases had a difference between intra-operative planned varus or valgus value and 1-year postoperative varus or valgus value of less than 3°.

### Clinical and functional outcomes

#### VAS

At 1-year postoperative assessment, the mean VAS was 1.6 ± 1.4 [1;4].

#### ROM

The mean flexion value was 133 ± 12 [100;140]. The mean extension value was 0.89 ± 2.1 [−5;5] no patient had knee extension deficit or recurvatum of more than 10°.

#### KOOS

A total of 69 patients (93%) had a good or excellent KOOS score (KOOS total > 70) at one year post-operative. KOOS score was excellent (KOOS total > 80) for 15 patients (20%) and good (between 70 and 80) for 54 patients (73%) (Table 2).

**Table 2:** KOOS subdivisions outcomes at one year postoperative.

KOOS subdivision (mean ± SD [min;max])	
KOOS total	77.75 ± 3.53 [65;84]
KOOS symptoms	82.67 ± 5.38 [65;94]
KOOS pain	78.41 ± 4.64 [69;88]
KOOS quality of life	74.28 ± 3.98 [59;81]
KOOS sport	70.74 ± 3.56 [65;81]
KOOS daily living	88.97 ± 4.15 [72;95]

KOOS: Knee Blessure and Osteoarthritis Outcome Score.

## Discussion

CT-based RATKA provides high-precision implant positioning for large deformities. Only one patient (1.4%) had a difference of more than 3° between the planned varus/valgus value and the 1-year post-operative varus/valgus value. This accuracy enables fitting to the desired alignment without compromising knee stability while giving the possibility of using semi-constrained implants. Functional results at one year postoperative were satisfying.

Our study is the only one to our knowledge to assess RATKA accuracy for the large preoperative deformity (more than 10° varus or valgus). It has been conducted on a continuous series of 74 RATKA performed by four senior surgeons on patients with preoperative large deformities (varus or valgus >10°). The procedure, alignment philosophy and implant type were the same for all patients included to limit bias.

Most of our patients had a difference of less than 3° between intra-operative varus or valgus planning and one-year post-operative measurements (98.6%). The mean difference was 0.6° for varus knees and 0.5° for valgus knees. The only outlier patient was in the valgus group and had a difference of 4° between intra-operative planning and post-operative measurement. This difference is slight and led to a final valgus of −5°. Those excellent accuracy are in favour of a real interest of RATKA for large deformities. No extra-articular bone procedures (osteotomy) had to be done to obtain optimal ligament balancing despite the large initial deformity. When necessary, a ligament release had to be performed but knee stability was not compromised, and all our patients have benefited from standard implants (semi-constrained) leading to better functional results and better long-term surveys in comparison with constrained implants [16, 17].

Our results show a reduction in cases with inappropriate post-operative alignment. The benefits of accurate prosthetic alignment for implant survival rates and functional results have been demonstrated [18, 19]. Our study’s follow-up does not enable us to demonstrate an improvement in implant survival. Long-term cohorts need to be performed to assess if RATKA affects long-term survival rates.

Our results echo prior studies in terms of outlier patients. [11, 20–22] Other studies have shown a higher difference between planned implant positioning and postoperative radiological measurements for conventional TKA in comparison with RATKA [10, 23]. A meta-analysis of Zhang et al. supports these findings. The mean difference between femoral or tibial component frontal positioning planification versus postoperative radiographic measurements was respectively 0.19° and 0.93° for the RATKA group versus 1.57 and 1.76 for the conventional TKA group [24].

Clinical outcomes and functional scores at one-year post-operative are good RATKA restores optimal joint kinematics and efficiently rectifies frontal deformities (knee extension deficit or recurvatum). Those good outcomes could be explained by the reduction of post-operative inflammatory reaction, reduction in soft tissues procedures or improvement in soft tissue protection already proved with RATKA procedures. Optimized ligament balance control also improves functional results by avoiding any overloading throughout the joint kinematic.

Literature is divergent about functional results after RATKA. Some studies have shown a superiority of RATKA in comparison with conventional TKA [24–26]. Batailler et al. showed in a meta-analysis the superiority of CT-based RATKA in terms of postoperative pain and functional outcomes at one year postoperative [27]. Other studies suggest more caution and found similar functional results between RATKA and conventional TKA [28, 29]. The absence of major alignment mistakes with RATKA certainly makes the poor results less severe than with conventional TKA, but comparative studies would be needed to confirm this hypothesis.

Alignment strategy probably contributes to both functional and radiological results. In our series, all implants were placed according to a patient-specific alignment (restricted kinematic alignment). Optimizing ligament balance probably improves reliability between alignment planned intra-operatively by the Mako system (off-weighting) and alignment measured post-operatively on long-leg standing X-rays (weight bearing). Ligament and tendon tensioning processes during weight bearing modify knee alignment, especially in cases of suboptimal ligament balancing. Patient-specific alignments reduce the need for ligament releases leading to better functional results. In their study evaluating the surgical factors that play a role in functional outcomes one year after RATKA Lundgren et al. showed that patient-specific alignment was a predictive variable to improve functional results [30]. In a study comparing functional alignment [31] and adjusted mechanical alignment with RATKA, Michaud et al. showed that functional alignment was superior to adjusted mechanical alignment in terms of implant positioning accuracy at one year post-operatively. Functional alignment had also short-term better functional results [32].

This study has some limitations. This is a prospective single cohort study with no matching group. A comparative study between RATKA and conventional TKA could have been more informative, but implants used for conventional TKA are not the same as those that are used for RATKA in our establishment. Group comparability would have been affected. The surgeon’s extensive experience in the field of TKA may have contributed to the good results. Manufacturer certification is required to use the Mako system, making it difficult to carry out the study on a more diversified panel of surgeons.

Post-operative varus or valgus values measurements have been done on X-ray. Precision is less important than with a CT scan post-operative measurement. It could not be done, because of the additional radiation exposure for the patient and the extra cost compared with conventional treatment.

This study focuses exclusively on CT-based RATKA. In a meta-analysis, Vermue et al. demonstrated that the type of robotic assistance may also influence functional and radiological outcomes [33]. Therefore, the conclusions can only be applied to this specific type of robotic assistance. Further studies with another kind of robotic assistance will be necessary to extend our conclusions to robotic assistance as a whole.

## Conclusion

For large preoperative deformities, CT-based RATKA provides a high degree of accuracy in implant positioning, permitting it to fit the desired alignment without compromising knee stability, and giving the possibility of using semi-constrained implants. At one-year postoperative functional results are encouraging and at this follow-up, most patients have recovered an optimal range of motions.

## Data Availability

This article has no associated data generated.
